# Impact of primary tumor volume and location on the prognosis of patients with locally recurrent nasopharyngeal carcinoma

**DOI:** 10.1186/s40880-015-0019-5

**Published:** 2015-06-10

**Authors:** Yun-Ming Tian, Wei-Wei Xiao, Li Bai, Xue-Wen Liu, Chong Zhao, Tai-Xiang Lu, Fei Han

**Affiliations:** Huizhou Municipal Central Hospital, Huizhou, Guangdong 516001 People’s Republic of China; Department of Radiation Oncology, Sun Yat-sen University Cancer Center; State Key Laboratory of Oncology in South China; Collaborative Innovation Center of Cancer Medicine, Guangzhou, Guangdong 510060 People’s Republic of China

**Keywords:** Nasopharyngeal carcinoma, Local recurrence, Prognostic value, Tumor location, Tumor volume

## Abstract

**Introduction:**

The properties of a tumor itself were considered the main factors determining the survival of patients with locally recurrent nasopharyngeal carcinoma (NPC) treated with intensity-modulated radiotherapy (IMRT). However, recurrent tumors were mainly evaluated by using the American Joint Committee on Cancer staging system, which was modeled on primary tumors and did not incorporate the tumor volume. This study aimed to investigate the prognostic values of the primary tumor location and tumor volume, and to determine whether evaluating these parameters could improve the current staging system.

**Methods:**

Magnetic resonance (MR) images for 229 patients with locally recurrent NPC who underwent IMRT were analyzed retrospectively.

**Results:**

The skull base, parapharyngeal space, and intracranial cavity were the most common sites of tumors. There was a difference in the survival between patients with T1 and T2 diseases (77.6 % vs. 50.0 %, *P* < 0.01) and those with T3 and T4 diseases (33.0 % vs. 18.0 %, *P* = 0.04) but no difference between patients with T2 and T3 diseases (50.0 % vs. 33.0 %, *P* = 0.18). Patients with a tumor volume ≤38 cm^3^ had a significantly higher survival rate compared with those with a tumor volume >38 cm^3^ (48.7 % vs. 15.2 %, *P* < 0.01).

**Conclusions:**

A new staging system has been proposed, with T3 tumors being down-staged to T2 and with the tumor volume being incorporated into the staging, which may lead to an improved evaluation of these tumors. This new system can be used to guide the treatment strategy for different risk groups of recurrent NPC.

## Background

Local control for nasopharyngeal carcinoma (NPC) has greatly improved with the development of radiotherapy and combined modality treatment; however, local recurrence still develops in patients with advanced-stage diseases [1–3]. Imaging plays an important role in detecting recurrence and becomes the only effective method of detection when the lesion is deeply seated. Compared with computed tomography (CT), magnetic resonance imaging (MRI) has better soft tissue contrast resolution and is superior in the determination of tumor boundaries. Moreover, MRI is a valuable method for distinguishing a recurrence from fibrosis caused by radiation [3–5].

NPC has a highly infiltrative nature, and as such, most recurrent cases are advanced. External beam radiation therapy is becoming the mainstay of treatment for recurrent NPC. However, compared with that of patients with primary tumors, the survival of patients with locally recurrent NPC after conventional radiotherapy is poor, owing to the decreased sensitivity to radiation and severe late complications [6–8]. Intensity-modulated radiotherapy (IMRT) targets the tumor site more effectively while minimizing the damage to adjacent organs and may improve the clinical outcome of patients with locally recurrent NPC [9–11]. Studies have shown that the properties of the tumor itself are the main factors determining the survival of patients treated with IMRT [10, 11]. In clinical practice, recurrent tumors are mainly evaluated according to the American Joint Committee on Cancer (AJCC) staging system; however, this system has several limitations. First, the current staging system was modeled on primary tumors, but recurrent NPCs differ in their natural behavior and therapeutic requirements. Second, the tumor volume has proven to be an important prognostic factor in recent research; however, it has not yet been incorporated into the staging system.

To address these issues, we assembled a large dataset from patients with locally recurrent NPC treated with IMRT and investigated the impact of the tumor sites and tumor volume on the survival by analyzing MRI. This study will allow for a more comprehensive evaluation of these tumors, which can, in turn, be used in guiding the treatment strategy for different risk groups of recurrent NPC.

## Patients and methods

### Patient selection

Between January 2001 and December 2008, 251 patients were diagnosed with locally recurrent NPC and underwent re-irradiation using IMRT in Sun Yat-sen University Cancer Center. A total of 229 patients were included in the study; the remaining 22 patients were excluded from the study, of which 19 had incomplete MRI information and 3 had distant metastasis prior to treatment. All participants provided written informed consent. Our protocol was approved by the institutional ethics committee of Sun Yat-sen University Cancer Center.

All 229 patients underwent MRI of the nasopharynx to evaluate the local tumor; chest X-ray examinations, sonography of the abdomen, and whole-body isotope bone scans were also carried out to detect any distant metastasis. Positron emission tomography-computed tomography (PET-CT) was also performed in 34 patients.

### MRI and the assessment of tumor sites

MRI examinations were performed by using a 1.5-T unit scanner (General Electric Medical Systems, Fairfield, CT, USA) with a combined head and neck coil. The following sequences were obtained before injecting the contrast material: T1-weighted images in the axial, coronal, and sagittal planes (repetition time, 500–600 ms; echo time, 10–20 ms) and T2-weighted images in the axial plane (repetition time, 4,000–6,000 ms; echo time, 95–110 ms). After a bolus injection of gadopentate dimeglumine (Gd-DTPA), contrast-enhanced T1-weighted images in the axial and sagittal planes and T1-weighted fat-suppressed coronal images were obtained.

Two radiologists specializing in head and neck cancers evaluated the MR images independently and graded tumors according to the 2009 AJCC staging system. Any disagreements were resolved by consensus according to the discussion of the team.

### IMRT and the measurement of tumor volume

All patients received a full course of IMRT. The target volumes were set according to the International Commission on Radiation Units and Measurements. A radiation oncologist manually outlined the gross tumor volume in the nasopharynx (GTV-nx) slice-by-slice on the pretreatment contrast-enhanced CT images in the planning system according to the MRI results. The tumor volume, equal to GTV-nx, was calculated by using the Corvus inverse IMRT planning system (version 3.0; Peacock 3.0, Nomos Corp, Sewickley, PA, USA) with the summation-of-area technique, which multiplies the area by the image reconstruction interval of 3 mm. The clinical target volumes (CTV) were designed to encompass the tumor (including the GTV) plus a 1.0- to 1.5-cm margin and a further small margin (<3 mm) where the tumor was in close proximity to critical intracranial structures or the spinal cord. An additional 2- to 3-mm margin was added to the CTV to create the planning target volume (PTV), allowing for setup variability and internal motion. The prescribed dose was 60–70 Gy to the GTV-nx and 50–54 Gy to the CTV, in 27–35 fractions. Treatment was delivered once daily, over 5 fractions per week. Cisplatin-based induction or concurrent chemotherapy was administered to 122 patients with recurrent T3–4 (rT3–4) and/or bulky gross tumors, including concurrent chemoradiotherapy to 46 patients, induction chemotherapy followed by radiotherapy to 63 patients, and induction and concurrent chemotherapy to 13 patients. The concurrent chemotherapy scheme was mainly cisplatin alone (80–100 mg/m^2^ intravenously every 21 days). The induction chemotherapy regimens included cisplatin (80–100 mg/m^2^ intravenously every 21 days) plus 5-fluorouracil (500 mg/m^2^ intravenously on days 1–5, every 21 days), and/or plus paclitaxel (175 mg/m^2^ intravenously every 21 days).

### Follow-up and statistical analysis

The duration of follow-up was calculated from the completion of IMRT to either the day of death or the date of the last examination. Patients were evaluated at least once every 3 months during the first 3 years and every 6 months thereafter until death. Severe late radiation-induced toxicities were recorded.

Receiver operating characteristic (ROC) curves were drawn to identify the cut-off point and test the prognostic validity of the tumor volume. The overall survival (OS), local failure–free survival (LFFS), and distant failure–free survival (DFFS) were calculated by using the Kaplan-Meier method, and the difference in survival curves was compared by using the log-rank test. The categorical variables, including the complications, were compared with the *χ*^2^ test. The level of significance was set at *P* < 0.05, and *P* values were based on two-sided tests.

## Results

### Patient characteristics

The patient cohort included 176 males and 53 females; the median age was 46 years (range, 21–79 years). The median interval from the end of primary radiotherapy to recurrence was 25 months (range, 6–248 months). Patients were diagnosed with local recurrence of NPC based on a biopsy (192 patients) or their clinical symptoms, signs, and imaging findings in cases of deep-seated lesions (37 patients). Using the 2009 AJCC staging system, the numbers of patients with stages I, II, III, and IVa diseases were 29, 28, 78, and 94, respectively. The majority of patients in the cohort (75.1 %) had advanced disease (stage III or IV).

### Tumor sites identified by MRI

**Fig. 1:**
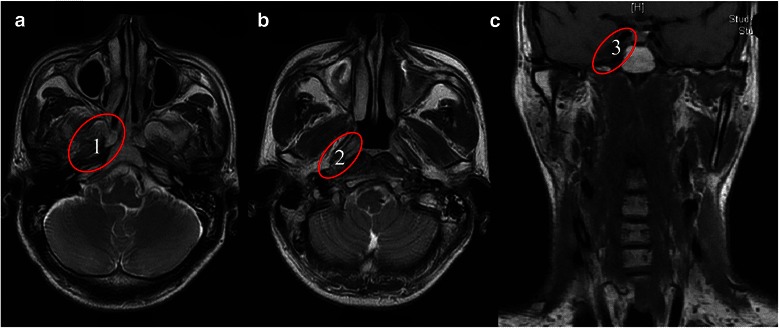
Magnetic resonance imaging findings of the common tumor invasion sites in patients with nasopharyngeal carcinoma (NPC). **a**, the skull base, the most common tumor invasion site (70.4 %); **b**, the parapharyngeal space (49.8 %); **c**, the intracranial cavity (32.3 %). The numbers in the pictures represent the order of incidence of tumor invasion

The frequency and location of tumor invasion sites identified by MRI are shown in Table 1. The most common tumor sites were the skull base (70.4 %; Fig. 1a), the parapharyngeal space (49.8 %; Fig. 1b), and the intracranial cavity (32.3 %; Fig. 1c). The tumor invasion routes to the cavernous sinus were via the foramen lacerum in 23 patients (32.4 %), via the foramen ovale in 19 patients (26.7 %), direct invasions via the clivus or sphenoid sinus in 12 patients (16.9 %), and via at least two of the aforementioned routes in 17 patients (23.9 %).

**Table 1:** The frequency and the sites of tumor invasion identified by magnetic resonance imaging in 229 patients with nasopharyngeal carcinoma (NPC)

Anatomical site	No. of patients (%)
**Skull base involvement**	162 (70.7)
Clivus	140 (61.1)
Basis of sphenoid bone	135 (59.0)
Petrous apex	120 (52.4)
Pterygoid process	114 (49.7)
Foramen lacerum	91 (40.0)
Great wing of sphenoid bone	59 (25.8)
Paranasal sinus	45 (19.7)
Foramen ovale	32 (13.9)
Pterygopalatine fossa	30 (13.1)
Jugular foramen	21 (9.7)
Hypoglossal canal	15 (6.5)
External auditory canal	6 (2.6)
**Soft tissue involvement**	147 (64.2)
Parapharyngeal space	114 (49.8)
Oropharynx	27 (11.8)
Nasal cavity	23 (10.4)
Prevertebral muscle	21 (9.2)
Masticator space	18 (7.9)
**Intracranial invasion**	74 (32.3)
Cavernous sinus	71 (31.0)
Retroclival space	11 (4.8)
**Orbital apex**	11 (4.8)

### Treatment outcome

The median follow-up time was 30 months (range, 5–147 months); 12 patients were lost to follow-up after 6 to 36 months (recorded as censored data). Forty-six patients (20.1 %) developed local failure and 28 (12.2 %) developed distant metastasis. The 5-year LFFS and DFFS rates were 70.8 % and 84.1 %, respectively.

A total of 155 patients in the cohort died, 71 of whom (45.8 %) died of radiation-induced injuries, 39 (25.2 %) died of local failures, 28 (18.0 %) died of distant failures, and 17 (11.0 %) died of unrelated causes. Of the 71 radiation-induced injuries, 36 were mucosa necrosis or massive hemorrhage, 13 were radiation-induced temporal lobe necrosis, and 22 were other radiation-related injuries. The 5-year OS rate was 36.6 %.

### Complications

**Fig. 2:**
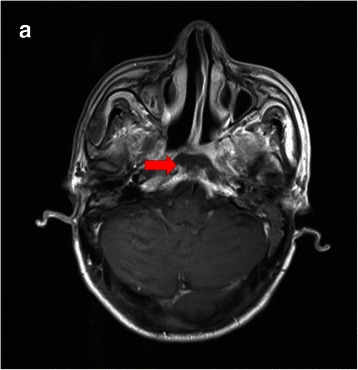
An NPC patient who developed severe mucosal and skull base necrosis after re-irradiation. The necrosis, indicating by the arrow, is near the internal carotid artery (ICA), an area more prone to developing massive hemorrhage

Most patients (85.9 %) developed mild to moderate acute toxicities while undergoing IMRT, and no patients had to stop the treatment. After completing IMRT, the following complications were observed: mucosal necrosis (77 of 229, 33.6 %; Fig. 2), temporal lobe necrosis (50 of 229, 21.8 %), massive hemorrhage (42 of 229, 18.3 %), and cranial neuropathy (28 of 229, 12.2 %). There was a significantly higher incidence of mucosal necrosis in patients with a tumor volume >38 cm^3^ compared with those with a tumor volume ≤38 cm^3^ (41.3 % vs. 27.2 %, *P* = 0.02). The tumor stage was also an independent factor predicting mucosal necrosis, with an incidence of 13.8 % in rT1, 32.1 % in rT2–3, and 41.5 % in rT4 cases (*P* = 0.04).

### Analysis of tumor sites and volume as prognostic factors

**Fig. 3:**
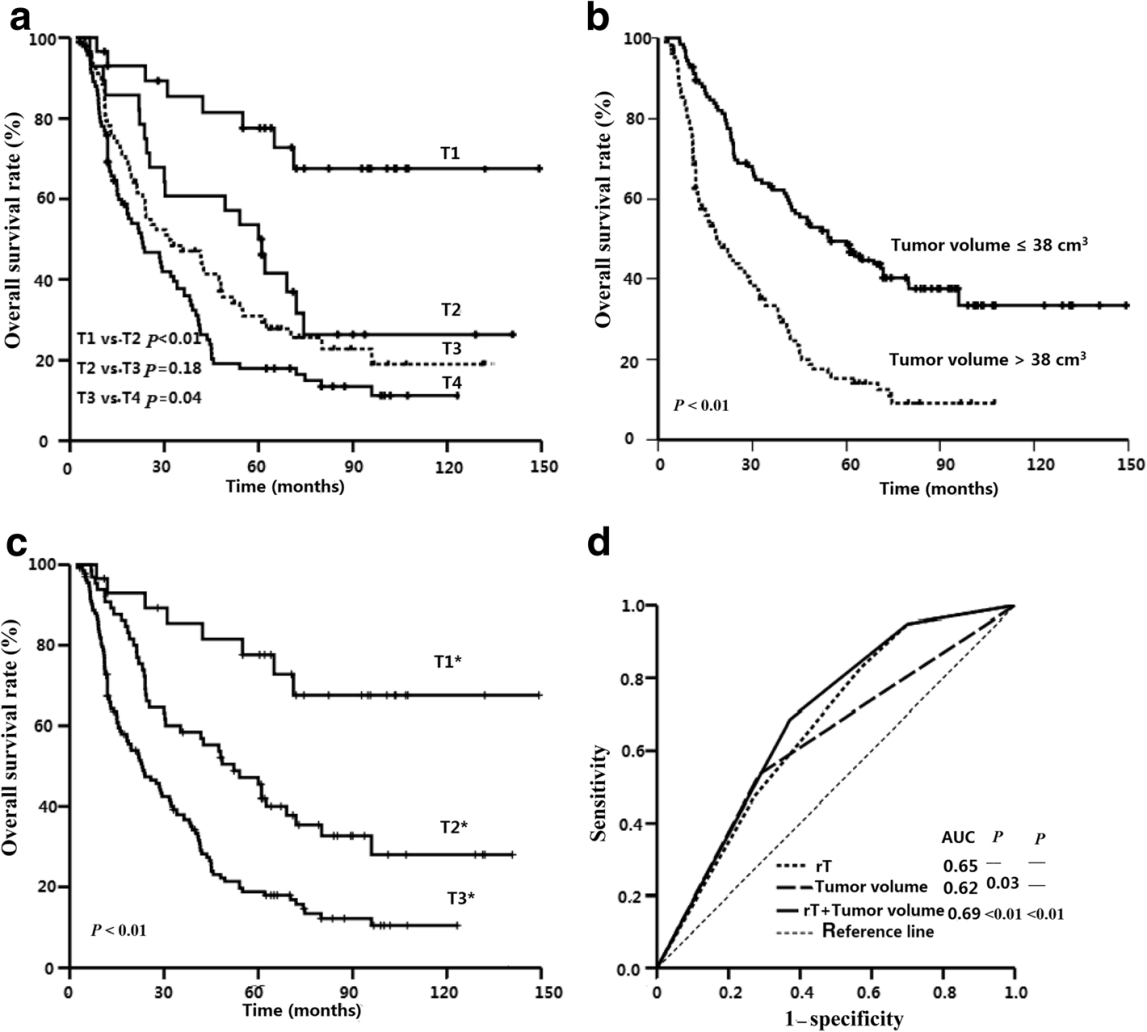
Survival curves for NPC patients with different factors. **a**, patients with the recurrent T category determined according to the American Joint Committee on Cancer (AJCC) staging system; **b**, patients stratified by the tumor volume; **c**, patients with the recurrent T category determined according to the proposed staging system (Tn*); **d**, receiver operator characteristic (ROC) curves for the recurrent T category alone, the tumor volume alone, and the addition of the tumor volume to the recurrent T category in the proposed staging system

Based on the invasion sites, recurrent tumors were evaluated according to the 2009 AJCC staging system. The 5-year OS rates for patients with T1, T2, T3, and T4 tumors were 77.6 %, 50.0 %, 33.0 %, and 18.0 %, respectively. The survival rates were significantly different between the patients with T1 and T2 tumors (*P* < 0.01) and between those with T3 and T4 tumors (*P* = 0.04). However, the difference in survival rates between the patients with T2 and T3 tumors was not significant (*P* = 0.18; Fig. 3a).

**Fig. 4:**
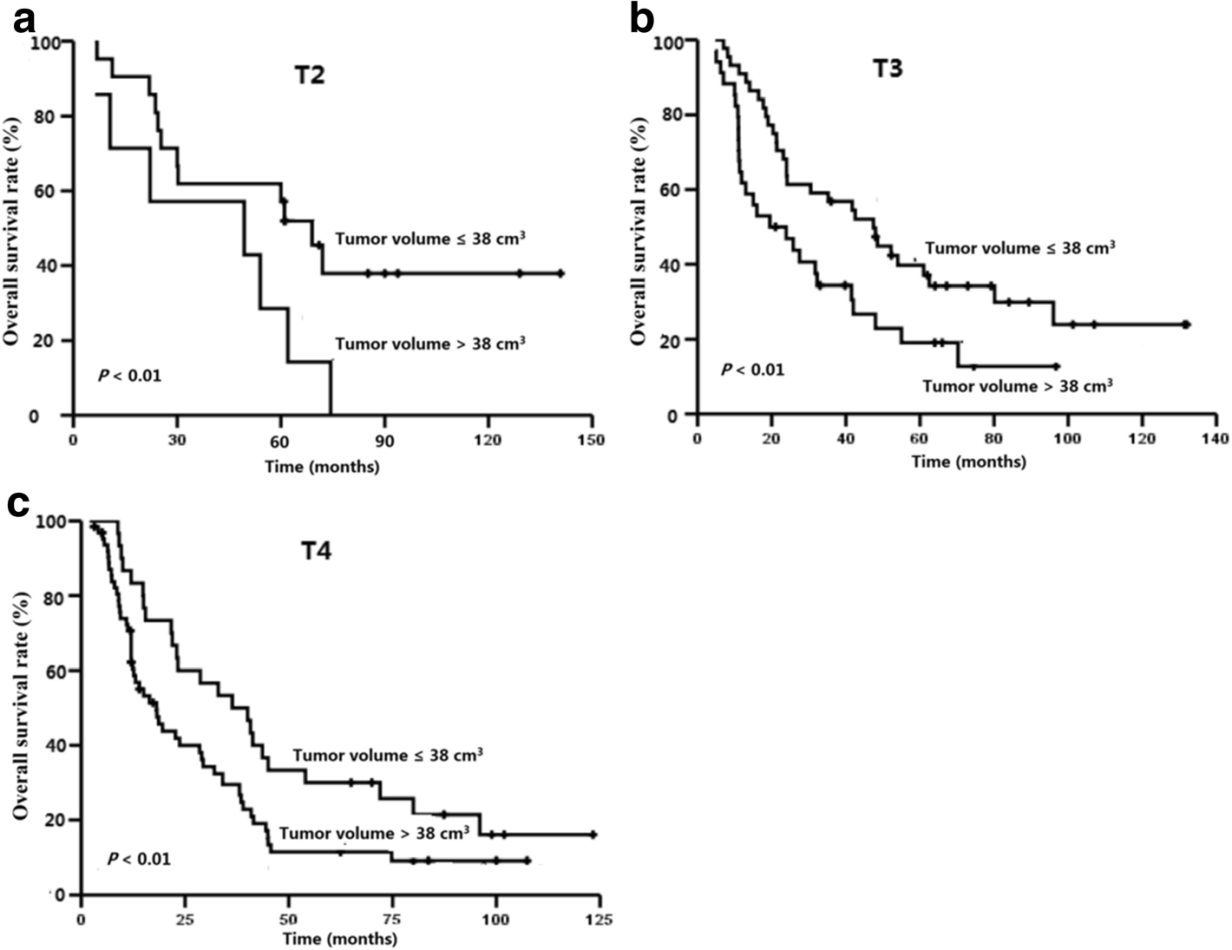
Survival curves for NPC patients with different tumor volumes in recurrent T2–4 groups. **a**, recurrent T2; **b**, recurrent T3; **c**, recurrent T4

The median tumor volume was 37.0 cm^3^ in the whole cohort; 11.4 cm^3^ (1.1–23.0 cm^3^) for T1 tumors, 25.4 cm^3^ (9.5–126.3 cm^3^) for T2 tumors, 35.0 cm^3^ (7.3–120.6 cm^3^) for T3 tumors, and 47.5 cm^3^ (8.7–146.4 cm^3^) for T4 tumors. The ROC curves showed that the cut-off value of tumor volume for the OS was 38.0 cm^3^. There was a significant difference in the survival rates between patients with a tumor volume ≤38 cm^3^ and patients with a tumor volume >38 cm^3^ (LFFS rate: 72.9 % vs. 56.9 %, *P* < 0.01; OS rate: 48.7 % vs. 15.2 %, *P* < 0.01, as shown in Fig. 3b). The tumor volume was also a prognostic factor for classifying patients with the same T category in a stratified analysis. The 5-year OS rates for patients with T2, T3, and T4 disease with a tumor volume ≤38 cm^3^ were 57.1 %, 39.7 %, and 19.1 %, respectively, whereas in patients with a tumor volume >38 cm^3^, these values were 28.6 % (*P* = 0.04), 19.1 % (*P* = 0.02), and 11.4 % (*P* = 0.01), respectively (Fig. 4).

The site(s) of tumor invasion and the tumor volume were therefore incorporated into a proposed new staging system for recurrent NPC (indicated by * in Table 2). Two revisions were made to the AJCC staging system based on the survival rates observed in this study: (1) T3 tumors were down-graded to T2, and both T2 and T3 categories were combined into T2*; and (2) all tumors with a volume >38 cm^3^ were classified into a high-risk group renamed T3*. The 5-year OS rates for the T1*, T2*, and T3* groups were 77.6 %, 45.5 %, and 18.8 %, respectively (*P* < 0.01; Fig. 3c). The OS curves for these groups were distinctly separated from each other. ROC curves were used to compare the prognostic value of incorporating the tumor volume into the T staging system. The area under the curve (AUC) was 0.65 for the T category alone, 0.62 for the tumor volume alone, and 0.69 for a combination of the two, indicating that the combination of the tumor sites and volume was superior to the T category alone (Fig. 3d).

**Table 2:** Tumor properties of NPC used to define the proposed new staging system

2009 AJCC staging system	Proposed new staging system
**T**: Tumor in the nasopharynx	T*: Tumor in the nasopharynx
**T1**: Tumor confined to the nasopharynx, nasal cavity, or oropharynx	T1*: As per AJCC T1 and with tumor volume ≤38 cm^3^
**T2**: Tumor with parapharyngeal extension	T2*: As per AJCC T2 and/or T3, and with tumor volume ≤38 cm^3^
**T3**: Tumor that invades bony structures and/or the paranasal sinuses	T3*: As per AJCC T4 and/or all tumors with a volume >38 cm^3^
**T4**: Tumor with intracranial extension and/or involvement of the cranial nerve, infratemporal fossa, hypopharynx, or orbit	

*Used to distinguish the tumor stage in the proposed new system from that in the American Joint Committee on Cancer (AJCC) staging system

## Discussion

In this retrospective study, MRI was used to evaluate the tumor size and location in patients with recurrent NPC, providing an accurate evaluation of tumor boundaries. Such accuracy is particularly important when using IMRT because it allows the specific targeting of tumor sites while sparing the surrounding tissues.

The skull base was demonstrated to be the most common site of extra-nasopharyngeal tumor invasion. This finding may be related to the propensity of the tumor for infiltration and the use of MRI, which is superior to CT scanning, in detecting minimal skull base invasion [12–14]. Consistent with the results of Chen et al. [14], we found no significant difference in the survival rates between patients with skull base invasion and those with T2 disease. This result could be due to the accuracy of MRI in detecting minimal bone marrow infiltration, leading to T category changes, or due to the use of IMRT, a treatment by which a more precise and higher radiation dose can be delivered specifically to the tumor to significantly improve local control. Furthermore, it was not always possible to distinguish minimal bone marrow infiltration from radiation-related changes, which led to difficulty in differentiating some cases of T3 disease from T2 disease. Therefore, it seemed appropriate to down-stage T3 to T2 and to combine them as T2* category; this strategy has also been recommended for primary NPC [15].

Intracranial invasion, including that of the cavernous sinus and retroclival space, was common in patients with recurrent NPC in this study. Using MRI with the contrast-enhanced fat-suppression technique can provide an accurate understanding of the route of extension into the intracranial cavity. We found that most recurrent tumors spread intracranially via the foramen lacerum and foramen ovale, which is consistent with that of perineural invasion [16]. Intracranial invasion is considered a negative prognostic factor because (1) tumors with intracranial invasion are associated with advanced and bulky diseases with poor local control [10, 11]; (2) the incidence of serious late complications, including mucosa necrosis and temporal lobe necrosis, increases with the size of the area that receives high-dose re-irradiation; and (3) these sites have anatomically rich venous plexuses and are conventionally thought to be potential routes of distant metastasis [17]. Therefore, the accurate diagnosis of intracranial invasion is important for creating an appropriate treatment plan.

The tumor volume has long been considered a significant prognostic factor for recurrent NPC [10, 11]. However, the data related to the role of the tumor volume in the staging system were limited. In this study, the cut-off point was evaluated by an ROC curve, and tumor volume (≤38 cm^3^ vs. >38 cm^3^) was shown to be an independent prognostic factor for OS. Patients with a large tumor (>38 cm^3^) were more likely to experience local failure and mucosal necrosis, which became the main challenge during re-irradiation using IMRT, and these patients had a low 5-year OS rate (15.2 %). Furthermore, the tumor volume was also a valuable factor in discriminating patients with T2–4 disease in the subgroup analysis. Therefore, we concluded that tumors with a volume >38 cm^3^ at any site should be considered part of the highest risk group (T3*). The incorporation of the tumor volume into the staging system for recurrent NPC can further improve the accuracy in predicting the prognosis and can be used to guide the treatment strategy for different risk groups.

The high incidence of late complications has become the main challenge for patients with recurrent NPC treated with IMRT. Mucosal necrosis can seriously affect the quality of life and lead to massive hemorrhage or even death. However, treatment can be complicated for the chronic nonhealing wound due to hypoxia and hypovascularity; these factors may be related to personal tolerance and the irradiation dose [18–20]. Temporal lobe necrosis is another complication that develops in patients following re-irradiation, and it occurred in 21.8 % of the patients in this study. To reduce the risk of late complications, it is critical to give an appropriate dose and identify the patients who will benefit most from re-irradiation to improve its therapeutic benefit.

## Conclusions

In conclusion, we have shown that the tumor location and volume were identified as independent prognostic factors for recurrent NPC. The proposed new staging system may lead to an improved evaluation of these tumors, which can be used to guide the treatment strategy for different risk groups of recurrent NPC. The proposed new staging system needs to be further tested and validated in a larger cohort of patients.
